# V.T.O.B.S.—Learning birth mechanics in virtual reality: a controlled cohort study in undergraduate medical education

**DOI:** 10.3389/fmed.2025.1715561

**Published:** 2025-11-12

**Authors:** Jana Adams, Christiane Klein, Sebastian Ludwig, Christoph Stosch, Kristina Vogel, Nicola H. Bauer, Christiane J. Bruns, Rabi R. Datta

**Affiliations:** 1 Department of Obstetrics, Faculty of Medicine and University Hospital Cologne, University of Cologne, Cologne, Germany; 2 Department of General-, Visceral-, Thoracic- and Transplant Surgery, Faculty of Medicine and University Hospital Cologne, University of Cologne, Cologne, Germany; 3 Department of Gynecology, Faculty of Medicine and University Hospital Cologne, University of Cologne, Cologne, Germany; 4 Cologne Interprofessional Skills-Lab and Simulation Centre, Faculty of Medicine and University Hospital Cologne, University of Cologne, Cologne, Germany; 5 Institute of Midwifery Science, Faculty of Medicine and University Hospital Cologne, University of Cologne, Cologne, Germany

**Keywords:** medical students, medical education, birth mechanics, obstetrics, virtual reality

## Abstract

**Introduction:**

Virtual reality (VR) is increasingly applied in medical education to enhance learning and patient care. Teaching birth mechanics poses particular challenges, as students must understand complex, dynamic, and rotating intrauterine processes that traditional models cannot adequately represent. VR offers immersive, interactive visualization and has shown promise in other fields, but its role in obstetrics remains underexplored. This study evaluated a novel VR module (Virtual Training for Obstetric Birth Simulation, V.T.O.B.S.) for undergraduate obstetrics education.

**Methods:**

In this single-center study, 46 medical students used V.T.O.B.S. during their obstetrical block internship and were compared with 120 students without VR exposure (non-equivalent intervention group design). The module consisted of a single, self-directed session. Knowledge retention was assessed 11–17 weeks later in a theoretical Objective Structured Clinical Examination (OSCE) station consisting mainly of image-based questions on birth mechanics. Secondary outcomes included acceptance, motion sickness, and free-text feedback.

**Results:**

No significant differences were found in long-term scores of the OSCE study station between VR and control groups. Subgroup analyses showed no consistent effects, except that visual impairment was associated with significantly lower performance (median 13 [IQR 11–15] vs. 14.5 [12–16], *p* = 0.003). Acceptance of VR was very high (median = 5 [IQR 4–5]), and motion sickness was rare and mild (median = 2 [1–3]). Free-text responses emphasized the innovative nature of the module, immersive 3D visualization, and the value of VR exposure in a university setting. Observed between-group effects were small and below the detectable threshold, suggesting that minor advantages may have remained uncaptured.

**Discussion:**

Although no significant knowledge gain was demonstrated, the strong acceptance and usability support the feasibility of VR in undergraduate obstetrics curricula. The absence of measurable effects may relate to the broad learning objective, brief OSCE assessment, single self-directed exposure, and heterogeneous timing between intervention and assessment. V.T.O.B.S. nonetheless represents an innovative educational tool addressing curricular gaps in visualizing dynamic birth mechanics. Future research should explore repeated exposures, assessment formats that directly capture spatial–conceptual understanding, and integration into interprofessional formats for medical and midwifery students as well as postgraduate training.

## 1 Introduction

The integration of modern teaching methods and innovative technologies is becoming increasingly important in advancing medical and midwifery education, ensuring high-quality patient care, and contributing to the continuous development of healthcare systems (1). Simulation-based training is widely recognized as an effective approach to enhance competence and confidence in obstetric care (2). National and international guidelines, including those of the German Society of Gynecology and Obstetrics (DGGG), emphasize the role of structured simulation training in improving interdisciplinary teamwork and performing invasive emergency procedures (3). However, realistic obstetric simulations remain rare. Organizational and financial constraints, limited access to simulation facilities, and the need to suspend clinical operations often result in such training being infrequent.

A sustainable understanding of birth mechanics has long been a challenge for students. Mastery requires not only solid anatomical knowledge of the maternal pelvis and the fetal skull but also the ability to integrate this with a 3D understanding of a complex, dynamic, and rotating process. For learners, spatial reasoning is particularly demanding, especially when processes are dynamic or rotational (4). Since birth mechanics occur within the maternal body, they are hidden from direct observation, further complicating comprehension. Traditional 3D models offer limited authenticity: fetal mannequins must be positioned manually by an instructor, and the maternal model is almost invariably depicted in the supine position, restricting the range of possible scenarios.

Virtual reality (VR) offers a promising solution to these limitations (5, 6). VR refers to a fully computer-generated, interactive environment experienced through head-mounted displays (HMDs) and, in many cases, additional input devices such as handheld controllers or gloves equipped with motion sensors (5, 7). Advanced tracking systems (either controller-based or through integrated hand-tracking cameras) allow the user's head, hands, and sometimes full body movements to be captured and mapped in real time into the virtual space. The field of view and stereoscopic display of modern HMDs provide a sense of depth, while spatial audio reproduces realistic soundscapes, both of which increase immersion.

From a pedagogical perspective, VR benefits from the principles of experiential learning: it allows learners to interact directly with dynamic, 3D content, repeat scenarios as often as needed, and receive immediate visual and auditory feedback (6, 8, 9). In medical education, VR has been shown to improve mental model accuracy, reduce cognitive load, and increase motivation and engagement (10, 11). Its strengths are particularly evident in areas requiring the visualization of anatomical structures, spatial relationships, and complex processes that are otherwise hidden from direct view, precisely the challenges encountered when teaching birth mechanics.

In obstetrics and gynecology, VR applications are still limited, often focusing on single topics such as cesarean section, neonatal resuscitation, or specific emergencies (12–14). Nonetheless, these studies demonstrate benefits in knowledge gain, skill acquisition, and confidence.

Recent research further underlines this trend, with several studies published between 2023 and 2025 demonstrating the growing role of immersive technologies for obstetric and surgical training. These include randomized trials on cesarean section and postpartum hemorrhage management (12, 15), VR-based emergency simulations for shoulder dystocia (13), and training of uterine balloon tamponade (14), all reporting improved procedural understanding and learner confidence. Together, these findings highlight the rapid technological and pedagogical advances that form the contemporary background for the present work.

In parallel, preliminary analyses from an ongoing national needs assessment conducted by our group (University of Cologne, 2024–2025) indicate that teaching professionals and students from both medicine and midwifery science perceive a high demand for a VR-based teaching tool capable of accurately depicting the physiology and pathology of birth mechanics.

Despite this growing evidence, only a few VR applications have been developed to support the understanding of pregnancy anatomy and labor mechanisms. For instance, Jones et al. (16) introduced a digital visualization of pregnancy anatomy, Ryan et al. (17) evaluated a VR-based learning module in midwifery education focusing on fetal development and labor processes, comparing immersive visualization with traditional lecture-based instruction and Hüseyinoglu and Yazici (18) recently tested a VR application for teaching the mechanism of labor in a randomized controlled trial. However, none of these programs provides a comprehensive and anatomically precise representation that encompasses both physiological and pathological mechanisms of labor, including fetal lie and position, engagement, rotation, and malpositions. V.T.O.B.S. was therefore designed to address this gap by enabling interprofessional training in a reproducible, location-independent, and loggable format, facilitating standardized learning and detailed debriefing. Such an approach has the potential to simplify and accelerate the acquisition of this complex knowledge, improve long-term retention, and ultimately contribute to safer obstetric practice. Despite these promises, evidence on the sustained learning effects of VR in medical education remains inconsistent, with most prior studies limited to short-term outcomes.

This study therefore aimed to develop and evaluate V.T.O.B.S., a novel VR-based module for teaching birth mechanics in undergraduate medical education. The primary endpoint was the long-term knowledge outcome measured in a theoretical OSCE station on birth mechanics. Secondary endpoints included user acceptance, motion sickness, and qualitative feedback.

## 2 Material and methods

The study was approved by the Ethics Committee of the Medical Faculty of the University of Cologne on 9/20/2024 (ref. number 24-1337). The study was registered upfront in the German Clinical Trials Register (DRKS) under the identifier DRKS00035188.

### 2.1 Hardware and *s*etup

**Figure 1:**
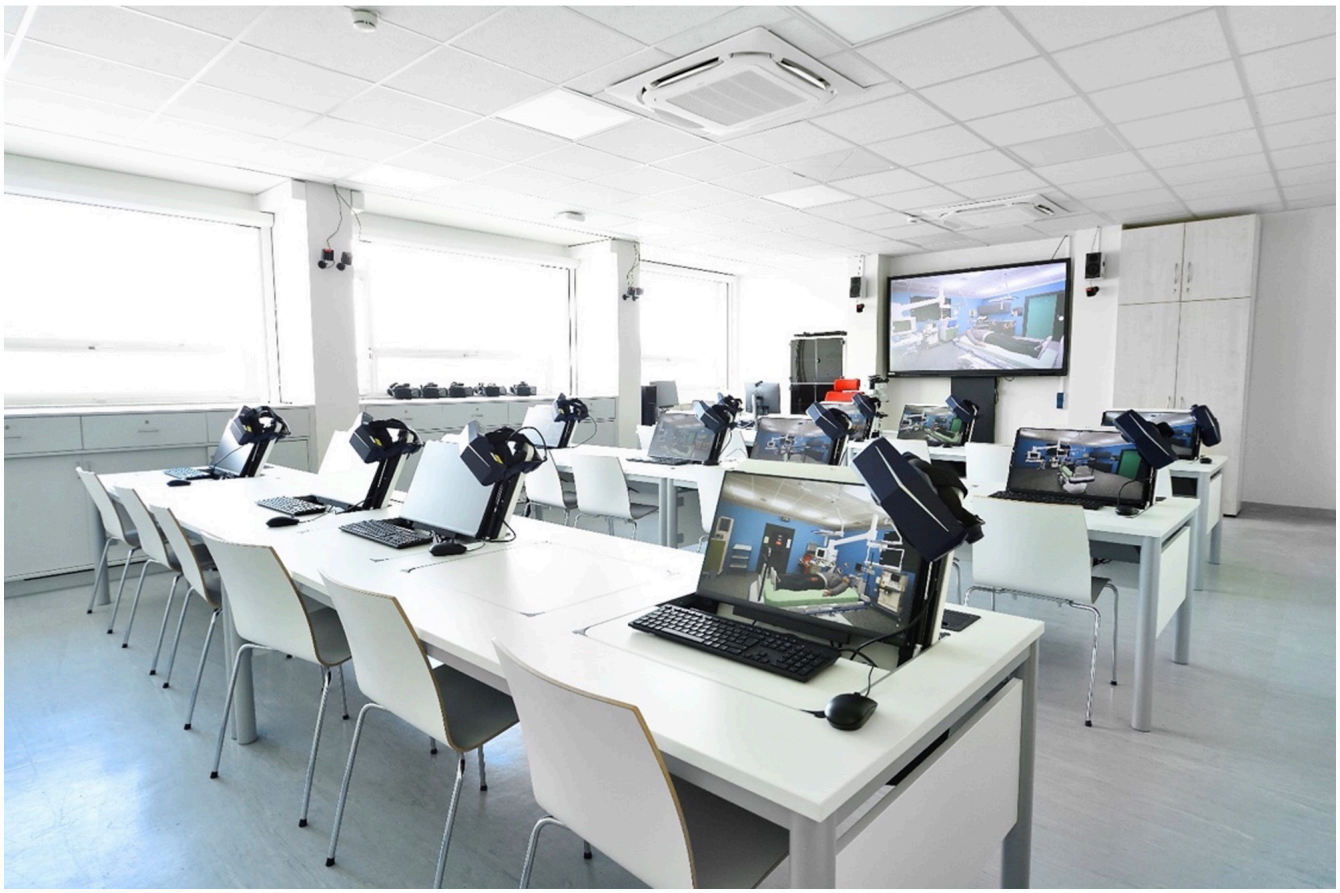
VR workplace set up at the Center for Medical Innovation and Technology (CeMIT).

The VR setup included an Alienware Aurora Ryzen Edition R10 Gaming Desktop and Pimax 8K Plus HMDs. Integrated hand tracking allowed for interaction without the need for handheld controllers. The intervention was conducted at the Center for Medical Innovation and Technology (CeMIT) on the campus of the University Hospital and University of Cologne, where 13 VR stations are available in parallel, allowing simultaneous training of larger student groups, as shown in Figure 1.

### 2.2 Virtual reality program

The VR application *Virtual Training for Obstetric Birth Simulation (V.T.O.B.S.)* was developed by the first author (JA) in collaboration with Marcus Mende, a programmer employed in our department and research group, and co-author Kristina Vogel (KV) from the Institute of Midwifery Science of the University of Cologne, using the UNITY engine. The project emerged from an interprofessional and was shaped through iterative feedback loops during development, which involved midwives as well as senior obstetricians.

**Figure 2:**
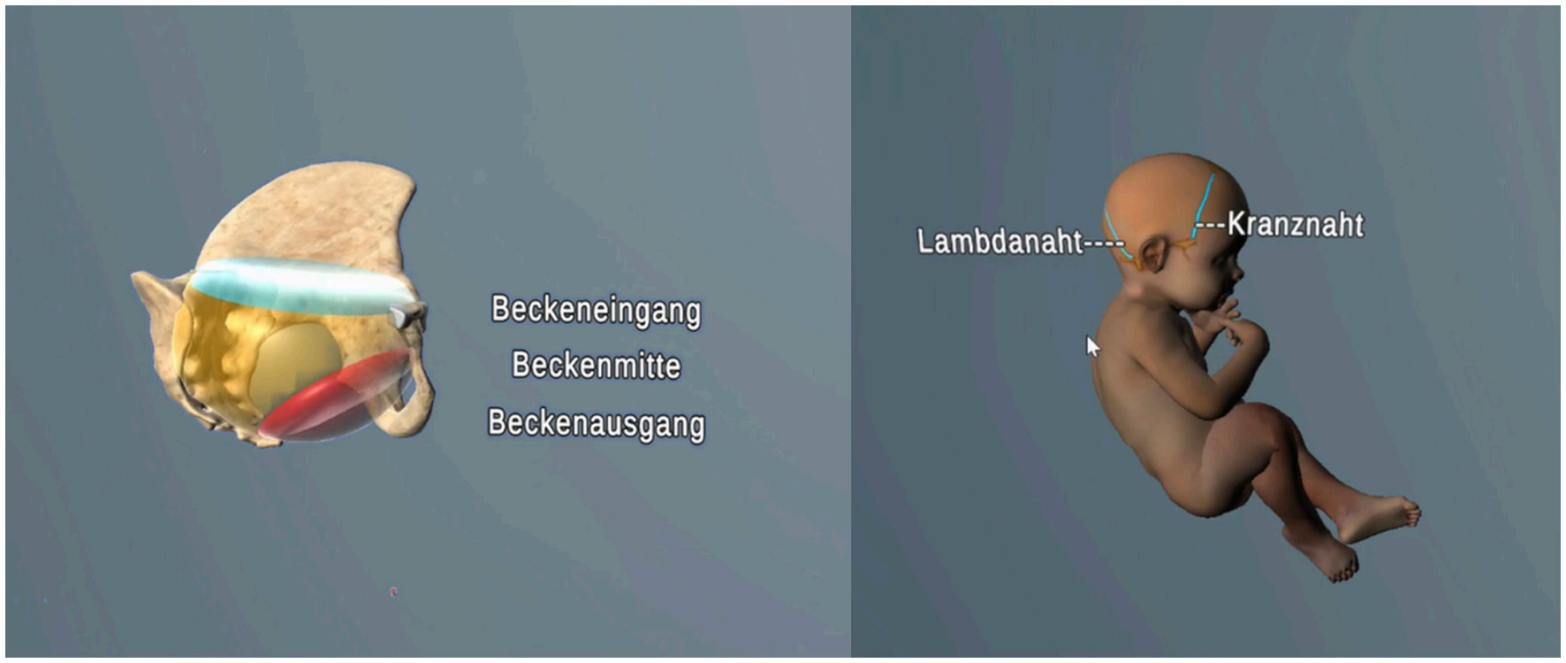
Visualization of the maternal bony pelvis and fetal cranial sutures during the introductory section (screenshot from V.T.O.B.S., in German).

Following a short technical introduction, the teaching module began with a review of anatomical basics related to the female pelvis and fetal skull, followed by an explanation of key obstetric terms used in birth mechanics (such as *lie, presentation*, and *position*) each illustrated with 3D examples of both physiological and pathological processes, as demonstrated in Figure 2.

**Figure 3:**
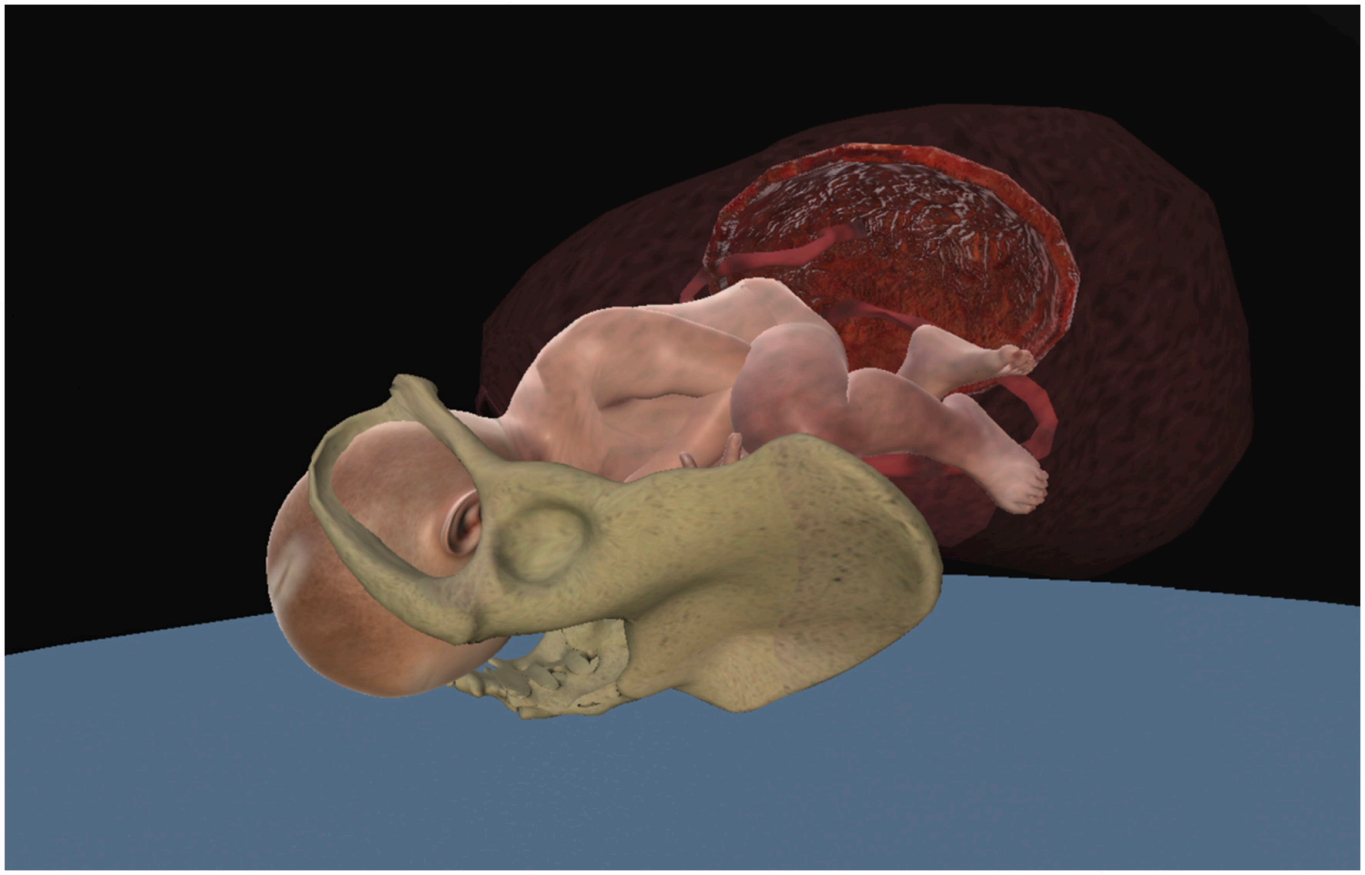
Anatomical model displaying the uterus, female pelvis and fetal-placental unit at approximately 10 × magnification (screenshot from V.T.O.B.S.).

**Figure 4:**
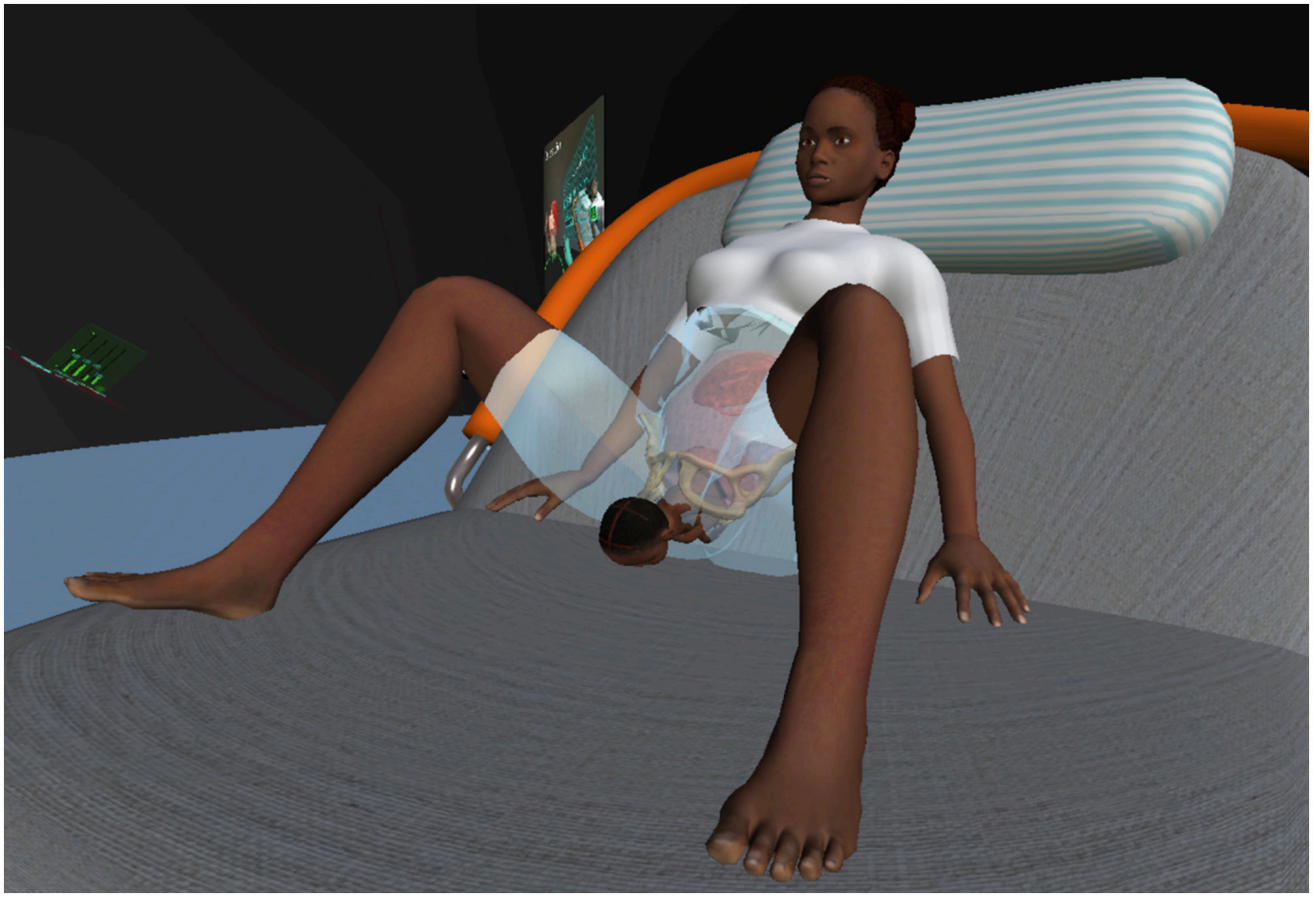
Full body model of a pregnant woman in labor (screenshot from V.T.O.B.S.).

Two virtual models were available to participants:

1. An isolated anatomical model displaying the uterus, female pelvis, and fetal–placental unit at approximately 10 × magnification, see Figure 3.
2. A full-body model of a pregnant woman in labor, whose body was transparent from the chest to the thighs, allowing visualization of bones, uterus, and fetal–placental unit, see Figure 4.

**Figure 5:**
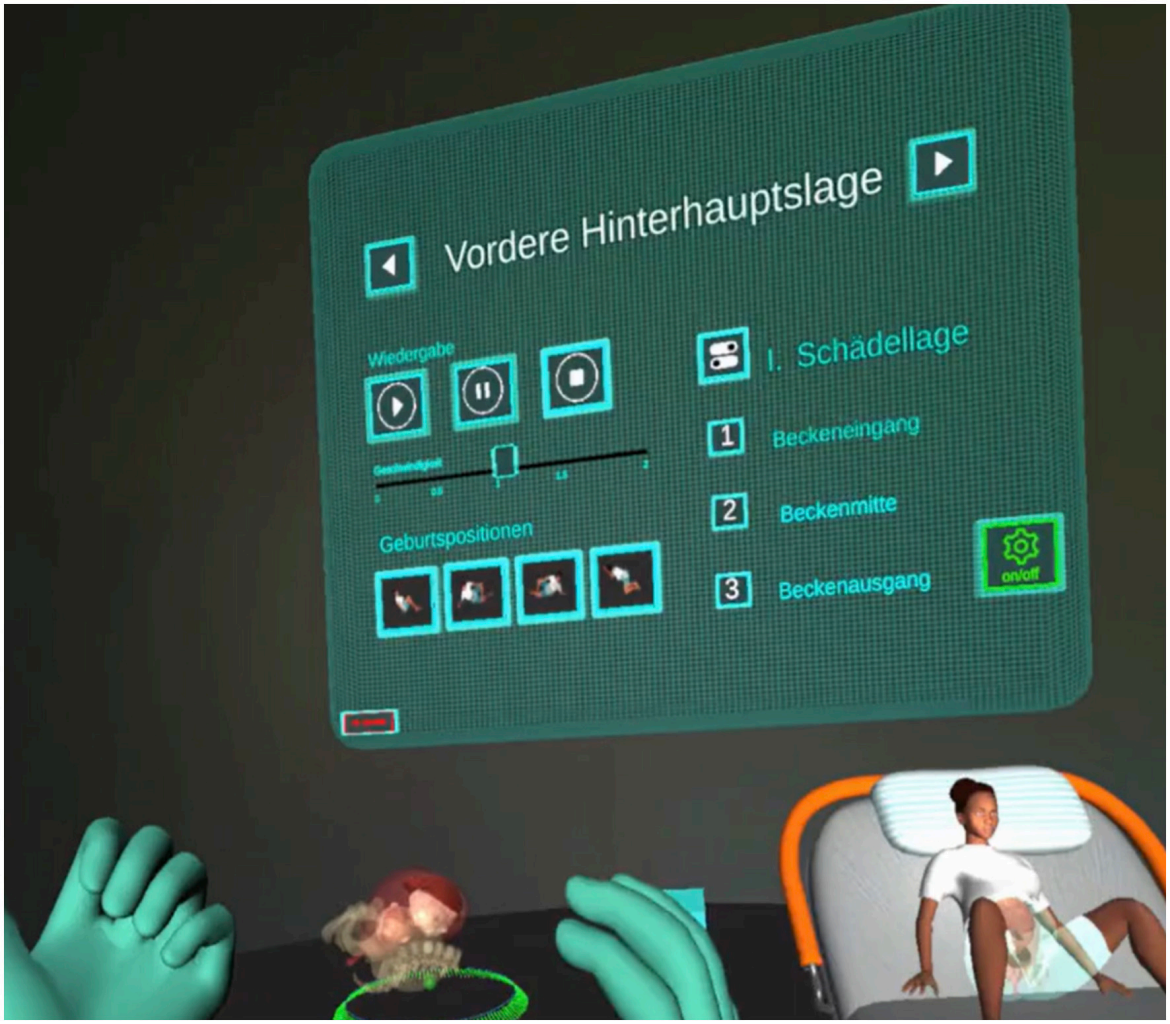
Control panel for selecting speed, pelvic level, maternal position and scenario/presentation (screenshot from V.T.O.B.S., in German).

Both models displayed fetal fontanelles and cranial sutures, whose visibility and bone density could be adjusted via a virtual control panel. Additional dashboard (see Figure 5) features allowed users to:

- Adjust the transparency of the uterine wall.
- Change the maternal position between supine, hands-and-knees, left lateral, and right lateral.
- Select the fetal position (occiput anterior, occiput posterior, or high straight position) in either the first or second stage of labor.
- Choose the starting point of the simulation at the pelvic inlet, mid-pelvis, or pelvic outlet.
- Adjust the speed of the birth process continuously, pause at any moment, and resume from the same point.

**Figure 6:**
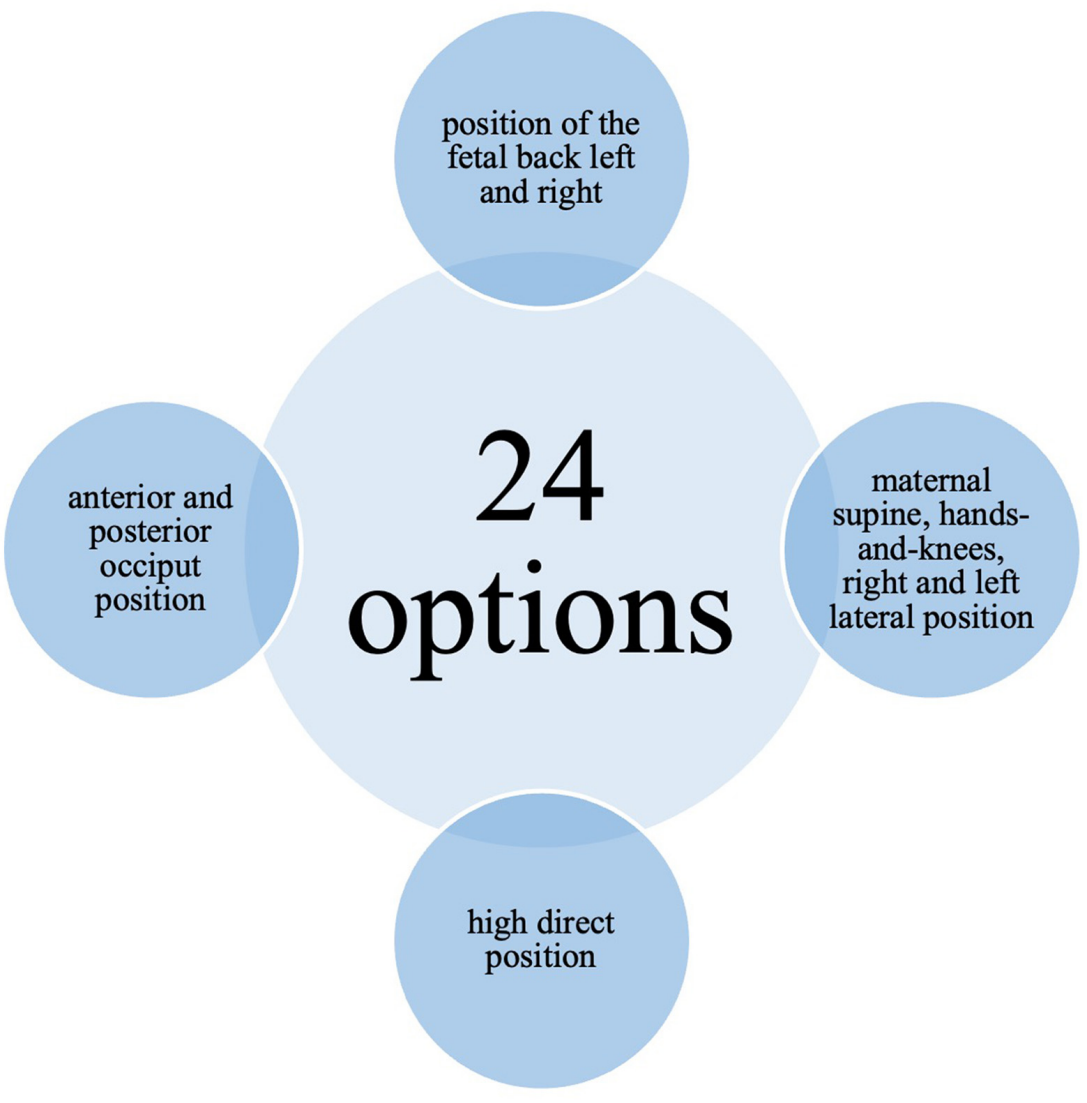
Possible combinations of visualized birth mechanical processes in V.T.O.B.S.

These settings produced 24 possible combinations of birth mechanical processes (see Figure 6). The two models could be switched at any time to view the same scenario from different perspectives. The full-body model allowed users to walk freely around the virtual delivery bed and observe the birth process from any angle, while the isolated uterine model could be rotated freely on its axis for detailed inspection.

### 2.3 Participants

Medical students of the 9th semester were recruited during their curricular block internship in gynecology and obstetrics. Students with non-correctable visual or motor deficits were defined as exclusion criteria. In total, 166 students participated in the study (46 in the intervention group, 120 in the control group). All participants had previously attended the lecture series on obstetrics and gynecology in the 7th semester and passed the corresponding exam. The basic principles of birth mechanics had already been covered in that context; therefore, the block internship content (for both intervention and control groups) was designed as a review. To reduce bias related to different time intervals between teaching and assessment, the control group included students from both the 8th and 9th semester, while the intervention group consisted exclusively of 9th-semester students.

### 2.4 Study design

After providing written informed consent, students in the 9th semester were randomly assigned either to the seminar group, which received the standard instructor-led teaching session on birth mechanics, or to the VR group, in which the conventional seminar was replaced by the V.T.O.B.S. module. Randomization was performed using sealed opaque envelopes and followed a single-blind procedure, as participants were not aware of their group allocation at the time of enrollment.

In addition, an 8th-semester cohort that had completed the block internship prior to the introduction of the VR module and received only the conventional seminar served as a non-randomized control group.

The block internship in gynecology and obstetrics took place between early October and the end of November 2024, while the Objective Structured Clinical Examination (OSCE) II was conducted at the end of the semester in late January 2025. Consequently, the time interval between the intervention and the knowledge assessment ranged from 11 to 17 weeks for both the intervention and the ninth-semester control group, whereas the eighth-semester control group completed the OSCE approximately 6 months after their internship.

Although the VR module was only implemented in the ninth semester, a small number of students (*n* = 7) indicated that they had participated in the VR intervention during the eighth semester. This discrepancy most likely reflects students who had attended the block internship ahead of schedule and also completed the OSCE examination early. These cases were therefore reassigned to the ninth semester VR intervention group. Consistent with this rationale, all analyses focused on the interval between the block internship and the OSCE rather than on the formal semester designation.

The control group attended a traditional, instructor-led session covering birth mechanics, while the VR group completed the V.T.O.B.S. module at the Center for Medical Innovation and Technology (CeMIT) at the University Hospital of Cologne. Both modules lasted up to 2 h, with most students completing the VR module within approximately 45–60 min.

### 2.5 Evaluation and outcome measures

Immediately after completing the VR session, the VR group filled out an evaluation form assessing demographic characteristics, prior VR experience, and preferred learning style. In addition, we included self-developed evaluation items to capture subjective user experience. Participants rated the statements “*I experienced motion sickness during the application*,” “*I perceived the virtual reality application as positive”* and “*The VR technology was useful for achieving the learning objectives*” on a 5-point Likert scale (1 = strongly disagree to 5 = strongly agree). Furthermore, two open-ended questions were included: “*What did you particularly like about the application?”* and “*What specific improvements would you suggest?”*

Knowledge outcomes were assessed at the end of the semester during the OSCE II. For this purpose, an additional, non-graded theory-based study station was integrated into the OSCE for all study groups. The station consisted of 12 questions (short-answer and multiple-choice format, including image-based items) with a total of 20 achievable points. Of these, five were image-based questions accounting for 10 points. Tasks included identifying birth mechanics in various positions and settings, one question on obstetric terminology, and one requiring the identification of fetal fontanelles. The station duration was 5 min.

The OSCE format was deliberately chosen as the outcome measure. In this study, the additional station was designed as a theory-based knowledge station, not a practical skills station, in order to assess conceptual understanding of birth mechanics. Since participation in OSCE II was mandatory for all students, integrating the study station minimized the risk of loss to follow-up. An alternative approach, such as inviting students to a voluntary post-test several weeks after the intervention, would likely have resulted in substantial drop-out and potential bias. In addition, the OSCE setting ensured standardized conditions and competency-oriented assessment, although the limited time frame of 5 min inevitably restricted the depth of testing. To account for the inherently spatial nature of birth mechanics, a high proportion of image-based tasks (five out of 12 items, worth 10 points) was included. This was intended to approximate the 3D reasoning processes required in clinical practice. Previous work has shown that image-based and visual–spatial tasks are effective in assessing anatomical and procedural understanding in OSCE contexts, particularly for domains involving spatial complexity such as anatomy and obstetrics (19, 20). By combining visual identification tasks with terminology and conceptual items, the station was designed to test both factual recall and the transfer of knowledge to schematic visual representations. The content validity of the station was further established by having it reviewed and completed by ten experienced obstetricians, ensuring that all items were clinically relevant and unambiguous.

### 2.6 Statistical analyses

No *a priori* sample size calculation was performed because the study comprised the complete cohort available within the curriculum (cross-sectional, curriculum-constrained design). To address this limitation, we conducted a sensitivity (power) analysis for the primary comparison (VR vs. control; Wilcoxon–Mann–Whitney, two-sided α = 0.05, 80% power). Given our group sizes (VR *n* = 46, control *n* = 120), the minimum detectable effect corresponded to AUC = 0.641, equivalently Cliff's δ = 0.283 and Cohen's *d* ≈ 0.51. This quantifies the smallest effect the study was sufficiently powered to detect. These non-parametric effect size measures were selected because they are well-suited for ordinal data and unequal group sizes, allowing meaningful interpretation of results even when normal distribution cannot be assumed.

Normal distribution was tested with the Kolmogorov–Smirnov test and not confirmed. Therefore, non-parametric procedures were applied. Between-group comparisons were performed using Mann–Whitney *U* tests for two groups and Kruskal–Wallis tests for three-group analyses. Correlations with ordinal variables (e.g., age, VR experience) were examined using Spearman's rank correlation. Nominal variables were tested with Mann–Whitney *U* (two groups) or Kruskal–Wallis (more than two groups). All analyses were performed with IBM^®^ SPSS Statistics version 30.0. A significance level of *p* < 0.05 was used. This conventional threshold aligns with current recommendations in biomedical and educational research (21).

## 3 Results

### 3.1 Demographics

**Figure 7:**
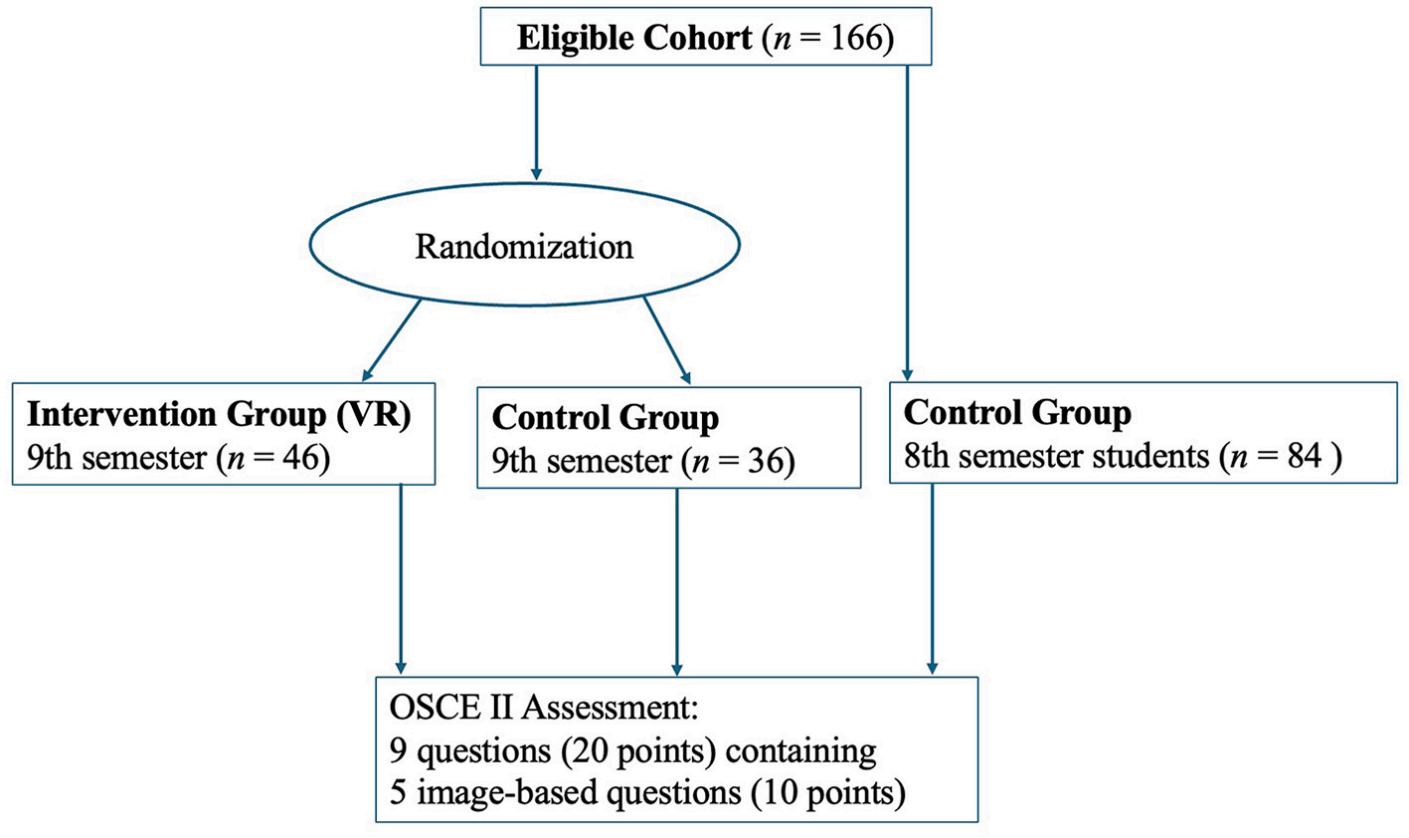
Study flow and group allocation.

A total of 166 medical students participated in the study (VR group: *n* = 46; control group: *n* = 120), see Figure 7. None met the exclusion criteria. Demographic characteristics are presented in Table 1. Unless otherwise stated, values are given as absolute numbers and percentages; age is reported as median with interquartile range [IQR].

**Table 1:** Participants demographic and background characteristics.

**Intervention (VR) group / Control group**	**Intervention (VR) group *n* = 46**	**Control group *n* = 120**
Age	25 [23–28]	26 [24–28]
**Gender**		
Male	11 (23.9%)	46 (38.2%)
Female	35 (76.1%)	73 (60.8%)
Divers	0	1 (0.8%)
**Semester of OB internship**		
8	0	84 (70%)
9	46 (100%)	36 (30%)
**Nationality**		
German	41 (89.1%)	118 (98.3%)
Other^a^	5 (10.9%)	2 (1.7%)
**Mother tongue**		
German	40 (87%)	116 (96.7%)
Other^b^	6 (13%)	4 (3.3%)
**German level**		
Native	40 (87%)	116 (96.7%)
C1	2 (4.3%)	2 (1.7%)
C2	4 (8.7%)	2 (1.7%)
**Visual impairment**		
No	23 (50%)	78 (65%)
Yes	23 (50%)	42 (35%)
**VR experience**		
No	20 (43.5)	69 (57.5%)
Yes	23 (50%)	44 (36.7%)
Missing	3 (6.5%)	7 (5.8%)
**Prior OB/GYN experience**		
No	37 (80.4%)	95 (79.2%)
Yes	9 (19.6%)	24 (20%)
Missing	0	1 (0.8%)
**Learning type** ^c^		
Visual	22 (13.3%)	72 (43.4%)
Non-visual	24 (14.5%)	48 (28.9%)
Auditory	9 (5.4%)	21 (12.7%)
Non-auditory	37 (22.3%)	99 (59.6%)
Read/write	33 (19.9%)	74 (44.6%)
Non-read/write	13 (7.8%)	46 (27.7%)
Kinesthetic	19 (11.4%)	49 (29.5%)
Non-kinesthetic	27 (16.3%)	71 (42.8%)

Count (percentage).

Age: Median (IQR).

^a^Includes nationalities represented only once (Bulgarian, Slovak, Luxembourgish, Irish, Korean, Turkish, Ukrainian).

^b^Includes mother tongues represented only once (Bulgarian, Slovak, Persian, Luxembourgish, Turkish, English, Ukrainian, Korean, Albanian).

^c^Percentages for *Learning type* do not sum to 100% because students could select more than one learning preference (multiple responses allowed).

Although some students indicated participation in the VR intervention during the 8th semester, the VR module was not implemented in that semester. These cases most likely reflect students who completed the internship and OSCE ahead of schedule. For methodological reasons, and to ensure comparability of the interval between internship and assessment, all VR participants were analyzed as part of the 9th semester VR group.

The median age was 25 years [IQR 23–28] in the VR group and 26 years [IQR 24–28] in the control group. The proportion of female students was higher in the VR group (76.1%) than in the control group (60.8%), while male participants accounted for 23.9% and 38.2%, respectively; one participant in the control group identified as diverse (0.8%).

Most students in the VR group completed their OB/GYN block internship in the ninth semester (84.8%), whereas the majority of control group students did so in the eighth semester (70%). A small number of students had completed their internship and OSCE ahead of schedule, which explains minor discrepancies in the semester distribution.

The majority of participants were of German nationality (VR 89.1%, control 98.3%); all other nationalities were represented only once and are summarized as “Other” (including Bulgarian, Slovak, Luxembourgish, Irish, Korean, Turkish, and Ukrainian). A similar pattern was seen for mother tongue, with German predominating (VR 87.0%, control 96.7%), and all other languages each represented by a single individual.

Most students reported German as their native language, but a small proportion had C1 (VR 4.3%, control 1.7%) or C2 (VR 8.7%, control 1.7%) proficiency. Approximately half of the VR group (50.0%) and one third of the control group (35.0%) reported having a visual impairment. Prior VR experience was more frequent in the VR group (50.0%) compared to the control group (36.7%). Previous gynecology experience was reported by about one fifth of participants in both groups (VR 19.6%, control 20.0%).

Self-reported learning styles were heterogeneous, multiple selections were possible. Among all 166 participants, 94 students (56.6%) indicated a visual learning preference, 30 (18.1%) an auditory preference, 107 (64.5%) a text-based (read/write) preference, and 68 (41%) a kinesthetic preference.

There were no statistically significant differences between the VR and control groups regarding demographic variables, with the exception of the semester in which the OB/GYN block internship was completed. This difference was due to the study design: the VR module was generally implemented in the ninth semester, while the control group primarily included students who had completed the internship in the eighth semester. A small number of VR group participants who indicated the eighth semester had likely attended the internship ahead of schedule. Subsequent analyses therefore focused on the comparison of learning outcomes and evaluation measures between groups. The following sections present the results of the performance assessment in the OSCE station, the subjective evaluation of the VR module, and the presence and motion sickness ratings.

Sensitivity analysis indicated that with *n* = 46 vs. *n* = 120 the design had 80% power to detect at least AUC = 0.641 (δ = 0.283; *d* ≈ 0.51). The observed effect corresponded to AUC = 0.544 (Cliff's δ = 0.088) with an approximate 95% CI for AUC of 0.445 to 0.642, consistent with no difference or effects smaller than the detectable threshold.

Because the OSCE total score and subscores were not normally distributed (Kolmogorov–Smirnov test, *p* < 0.05), all group comparisons were conducted using non-parametric tests. Mann–Whitney *U* tests were applied for comparisons between two groups, and Kruskal–Wallis tests for comparisons involving more than two groups. Results are presented as median [IQR] together with mean ranks and corresponding *p*-values.

### 3.2 Overall performance

The primary outcome measure was the total score of the OSCE study station, consisting of 12 questions with a maximum of 20 achievable points. The questions covered different aspects of birth mechanics, including terminology, recognition of fetal fontanelles, and interpretation of various positions and settings. In addition, a secondary outcome measure was defined as the image-based score, which included all items accompanied by visual material (images and image-based follow-up questions). This subscore comprised five questions with a maximum of 10 achievable points.

**Figure 8:**
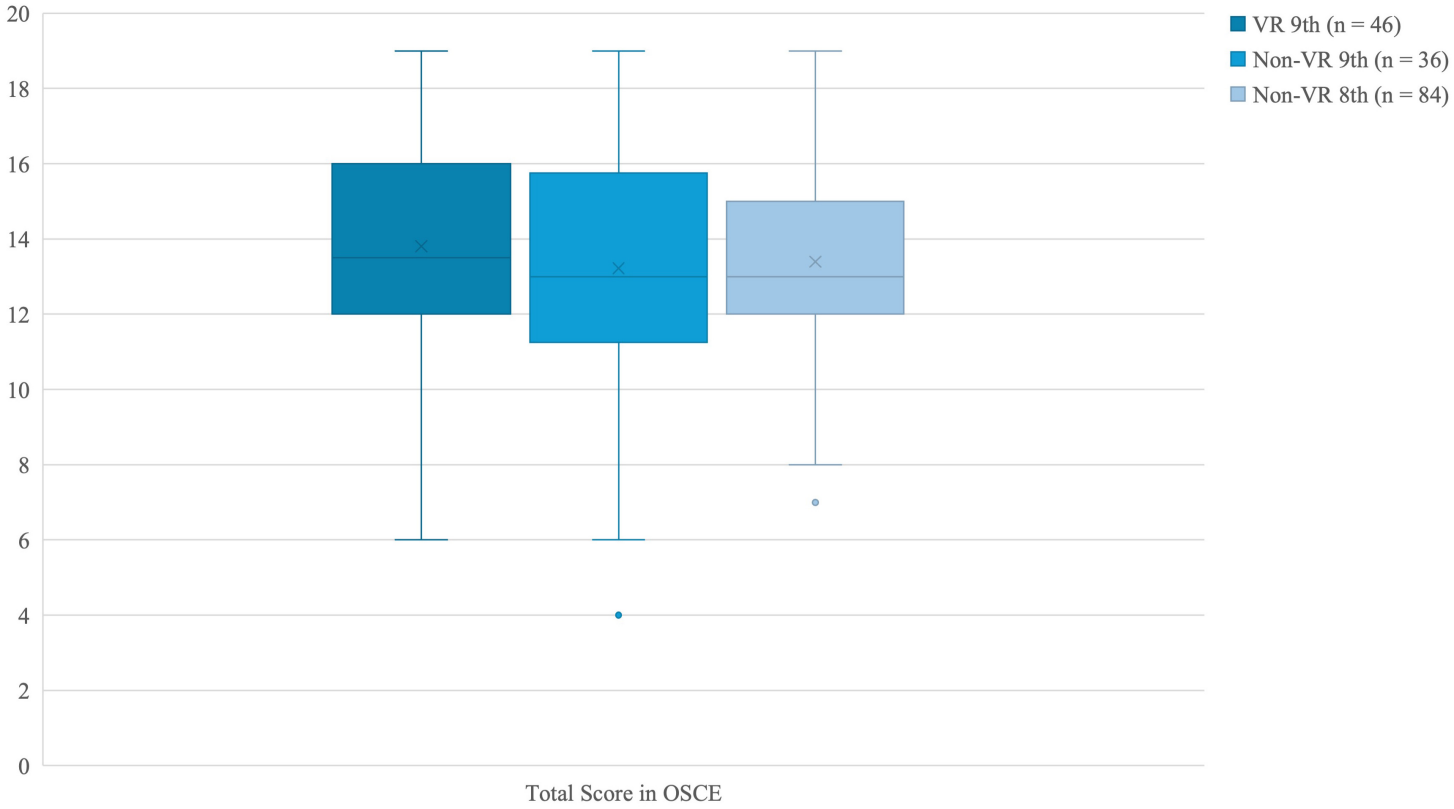
Boxplots of OSCE total scores (maximum of 20 points; median and interquartile range) for students in the VR and non-VR groups (9th semester) and the non-VR group (8th semester). Group comparisons were performed using the Kruskal–Wallis test.

The median total OSCE score did not differ significantly between students who participated in the VR intervention and those in the non-VR groups (13.5 [12–16] vs. 13 [12–15], *p* = 0.380, Mann–Whitney *U*; Table 2). Likewise, no significant differences were found between eighth- and ninth-semester students (13 [11–15] vs. 13 [12–16], *p* = 0.371). In the four-group comparison (VR 9th, non-VR 9th, VR 8th, and non-VR 8th semester), no statistically significant differences were detected (*p* = 0.521, Kruskal–Wallis), although ninth-semester VR students reached the highest median score (14 [13–16]), as demonstrated in Figure 8.

**Table 2:** Performance in OSCE station (total score and image-based subscores).

**Comparison**	**Groups**	**Median [IQR]**	**Mean rank**	***p*-Value**
**Total score (max. 20 points)**				
VR	VR (*n* = 46)	13.5 [12.0–16.0]	88.8	0.380^a^
	Non-VR (*n* = 120)	13.0 [12.0–15.0]	81.5	
Semester	8th (*n* = 84)	13.0 [12.0–15.0]	81.5	0.583^a^
	9th (*n* = 82)	13.0 [12.0–16.0]	85.6	
Combined	VR 9th (*n* = 46)	13.5 [12.0–16.0]	88.8	0.681^b^
	Non-VR 9th (*n* = 36)	13.0 [11.3–15.8]	81.5	
	Non-VR 8th (*n* = 84)	13.0 [12.0–15.0]	81.5	
**Image-based questions (max. 10 points)**				
VR	VR (*n* = 46)	7.0 [5.0–8.0]	82.0	0.797^a^
	Non-VR (*n* = 120)	7.0 [4.3–8.0]	84.1	
Semester	8th (*n* = 84)	6.5 [4.3–8.0]	84.3	0.835^a^
	9th (*n* = 82)	7.0 [5.0–8.0]	82.7	
Combined	VR 9th (*n* = 46)	7.0 [5.0–8.0]	82.0	0.966^b^
	Non-VR 9th (*n* = 36)	7.0 [4.3–8.0]	83.7	
	Non-VR 8th (*n* = 84)	6.5 [4.3–8.0]	84.3	

^a^Mann–Whitney U test.

^b^Kruskal–Wallis test.

Descriptive statistics are rounded to one decimal for presentation; all statistical tests were computed on unrounded values. Minor discrepancies reflect rounding.

**Figure 9:**
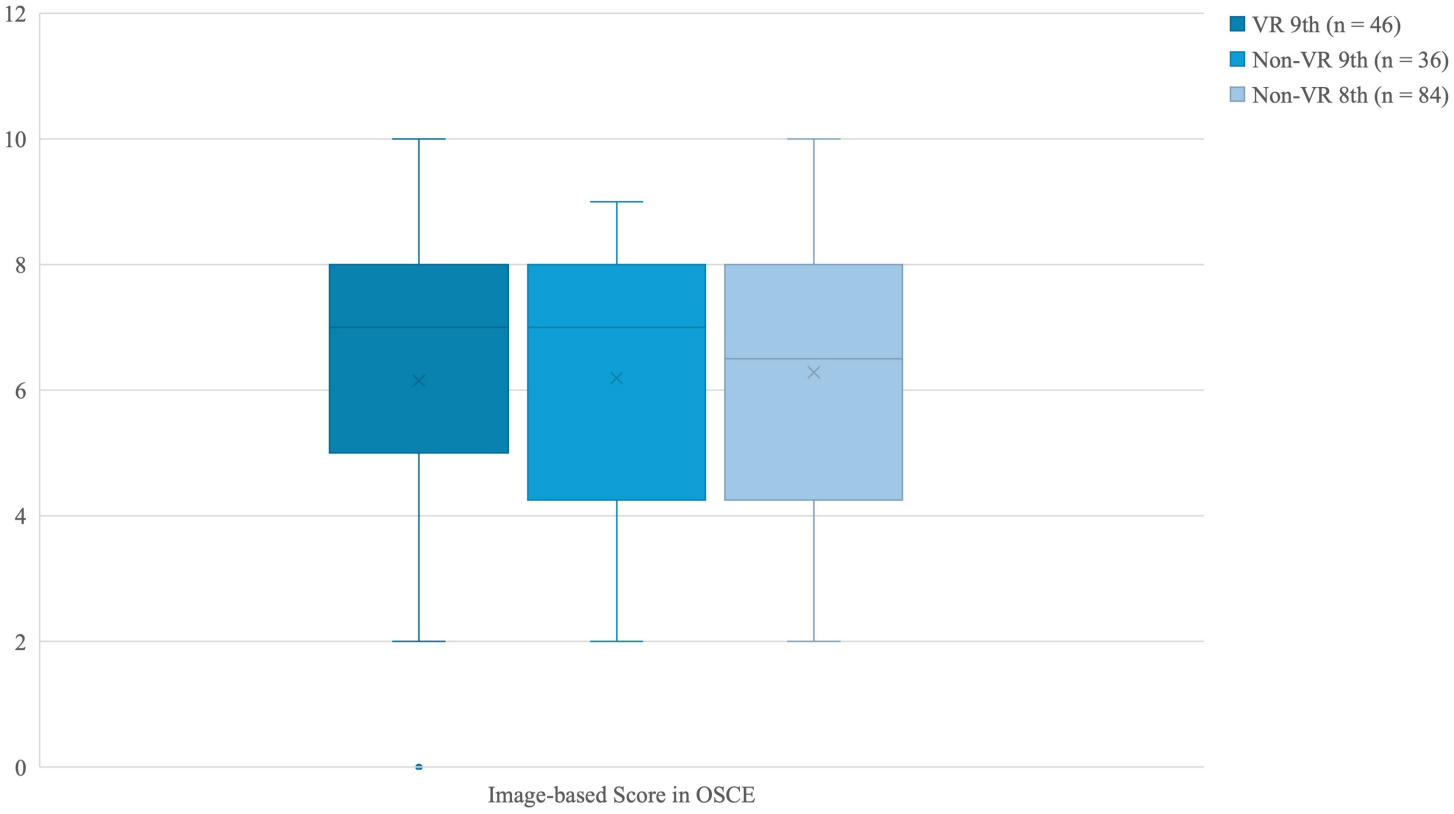
Boxplots of OSCE Image-based scores (maximum of 10 points; median and interquartile range) for students in the VR and non-VR groups (9th semester) and the non-VR group (8th semester). Group comparisons were performed using the Kruskal–Wallis test.

For image-based questions, no significant group differences were observed (VR vs. non-VR: 7 [5–8] vs. 7 [4.25–8], *p* = 0.797; eighth vs. ninth semester: 7 [4–8] vs. 7 [5–8], *p* = 0.885). The four-group analysis again showed no significance (*p* = 0.856), with VR students in the eighth semester performing lowest and non-VR students in the eighth semester highest (Table 2), as demonstrated in Figure 9.

### 3.3 Subgroup analyses

Gender did not significantly influence OSCE performance (male 14 [12–16] vs. female 13 [12–15], *p* = 0.263, Mann–Whitney *U*; Table 3). In the combined gender × VR comparison, no significant differences emerged (*p* = 0.497). Similarly, prior VR experience (*p* = 0.971) and prior gynecology/obstetrics experience (*p* = 0.949) were not associated with significant performance differences. However, in the combined analyses, ninth-semester VR students without prior VR experience achieved the highest scores, and VR students with prior gynecology experience also tended to score higher, though these effects did not reach significance.

**Table 3:** Subanalyses for gender, prior VR experience, prior OB/GYN experience, visual impairment with VR or NON-VR in correlation with total scores in OSCE station.

**Comparison**	**Groups**	**Median [IQR]**	**Mean rank**	***p*-Value**
Gender	Male (*n* = 57)	14.0 [12.0–16.0]	88.7	0.263^a^
	Female (*n* = 108)	13.0 [12.0–15.0]	80.0	
Combined	Male + VR (*n* = 11)	14.0 [13.0–16.0]	98.5	0.497^b^
	Male + Non-VR (*n* = 46)	13.5 [12.0–16.0]	86.4	
	Female + VR (*n* = 35)	13.0 [12.0–16.0]	84.9	
	Female + Non-VR (*n* = 73)	13.0 [11.0–15.0]	77.6	
Prior VR experience	Yes (*n* = 67)	13.0 [12.0–15.0]	78.67	0.971^a^
	No (*n* = 89)	13.0 [11.5–16.0]	78.4	
Combined	VR + VR experience (*n* = 23)	13.0 [12.0–15.0]	72.8	0.189^b^
	Non-VR + VR experience (*n* = 44)	13.0 [12.0–15.8]	81.7	
	VR + no VR experience (*n* = 20)	15.5 [13.0–16.0]	96.5	
	Non-VR + no VR Experience (n = 69)	13.0 [11.0–15.0]	73.2	
Prior OB/GYN experience	Yes (*n* = 33)	13.0 [12.0–16.0]	83.5	0.949^a^
	No (*n* = 132)	13.0 [12.0–15.0]	82.9	
Combined	VR + OB/GYN experience (*n* = 9)	16.0 [12.5–19.0]	115.1	0.134^b^
	Non-VR + OB/GYN experience (*n* = 24)	12.0 [11.0–15.0]	71.6	
	VR + no OB/GYN experience (*n* = 37)	13.0 [12.0–15.0]	81.1	
	Non-VR + no OB/GYN Experience (*n* = 95)	13.0 [12.0–15.0]	83.6	
Visual impairment	Yes (*n* = 87)	13.0 [11.0–15.0]	72.5	0.003^*^
	No (*n* = 78)	14.5 [12.0–16.0]	94.8	
Combined	VR + visual impairment (*n* = 29)	13.0 [12.0–15.5]	83.2	0.010^*^
	Non-VR + visual impairment (*n* = 58)	13.0 [10.0–15.0]	67.1	
	VR + no visual impairment (*n* = 16)	14.5 [12.3–16.0]	98.0	
	Non-VR + no visual impairment (*n* = 62)	14.5 [12.0–16.0]	93.9	

^a^Mann–Whitney U test.

^b^Kruskal–Wallis test.

^*^Indicating significancy.

Descriptive statistics are rounded to one decimal for presentation; all statistical tests were computed on unrounded values. Minor discrepancies reflect rounding.

Visual impairment was the only variable showing a statistically significant effect: students without visual impairment scored higher overall (14.5 [12–16] vs. 13 [11–15], *p* = 0.003, Mann–Whitney *U*). In the combined analysis, VR students without visual impairment achieved the highest median score (14.5 [12–16]), whereas non-VR students with visual impairment performed lowest (*p* = 0.010, Kruskal–Wallis; Table 3).

### 3.4 Learning types

No significant associations were observed between self-reported learning styles and OSCE performance (Table 4). Across all four learning types (visual, auditory, text-based, and kinesthetic), neither the main effect (yes/no) nor the combined analyses with VR vs. non-VR groups yielded significant results (all *p* > 0.2). Small, non-significant tendencies suggested slightly higher scores for kinesthetic learners and for students who identified as text learners without VR exposure.

**Table 4:** Subanalyses for learning types with VR or Non-VR in correlation with total scores in OSCE station.

**Comparison**	**Groups**	**Median [IQR]**	**Mean rank**	***p*-Value**
Visual learner	Yes (*n* = 94)	13.0 [12.0–15.0]	83.1	0.902^a^
	No (*n* = 72)	13.0 [12.0–16.0]	84.0	
Combined	VR + visual learner (*n* = 22)	3.0 [12.0–16.0]	88.6	0.857^b^
	Non-VR + visual learner (*n* = 72)	13.5 [12.0–15.0]	81.4	
	VR + non-visual learner (*n* = 24)	14.0 [12.3–15.8]	88.9	
	Non-VR + non- visual learner (*n* = 48)	13.0 [11.3–16.0]	81.6	
Auditory learner	Yes (*n* = 30)	14.0 [12.0–15.3]	86.6	0.696^a^
	No (*n* = 136)	13.0 [12.0–15.0]	82.8	
Combined	VR + auditory learner (*n* = 9)	13.0 [12.0–16.5]	92.8	0.822^b^
	Non-VR + auditory learner (*n* = 21)	14.0 [11.5–15.0]	83.9	
	VR + non-auditory learner (*n* = 37)	14.0 [12.0–16.0]	87.8	
	Non-VR + non-auditory learner (*n* = 99)	13.0 [12.0–15.0]	81.0	
Text-based Learner	Yes (*n* = 107)	13.0 [11.0–16.0]	82.3	0.656^a^
	No (*n* = 59)	13.0 [12.0–15.0]	85.7	
Combined	VR + text learner (*n* = 33)	13.0 [12.0–15.0]	80.5	0.217^b^
	Non-VR + text learner (*n* = 74)	13.5 [11.0–16.0]	83.0	
	VR + non-text learner (*n* = 13)	14.0 [13.5–16.5]	109.7	
	Non-VR + non-text learner (*n* = 46)	13.0 [12.0–15.0]	79.0	
Kinesthetic Learner	Yes (*n* = 68)	14.0 [12.0–15.0]	86.6	0.486^a^
	No (*n* = 98)	13.0 [11.0–15.3]	81.4	
Combined	VR + kinesthetic learner (*n* = 19)	14.0 [13.0–16.0]	101.4	0.383^b^
	Non-VR + kinesthetic learner (*n* = 49)	13.0 [12.0–15.0]	80.9	
	VR + non-kinesthetic learner (*n* = 27)	13.0 [12.0–15.0]	79.9	
	Non-VR + non-kinesthetic learner (*n* = 71)	13.0 [11.0–16.0]	81.9	

^a^Mann–Whitney U test.

^b^Kruskal–Wallis test.

Descriptive statistics are rounded to one decimal for presentation; all statistical tests were computed on unrounded values. Minor discrepancies reflect rounding.

### 3.5 Evaluation

**Figure 10:**
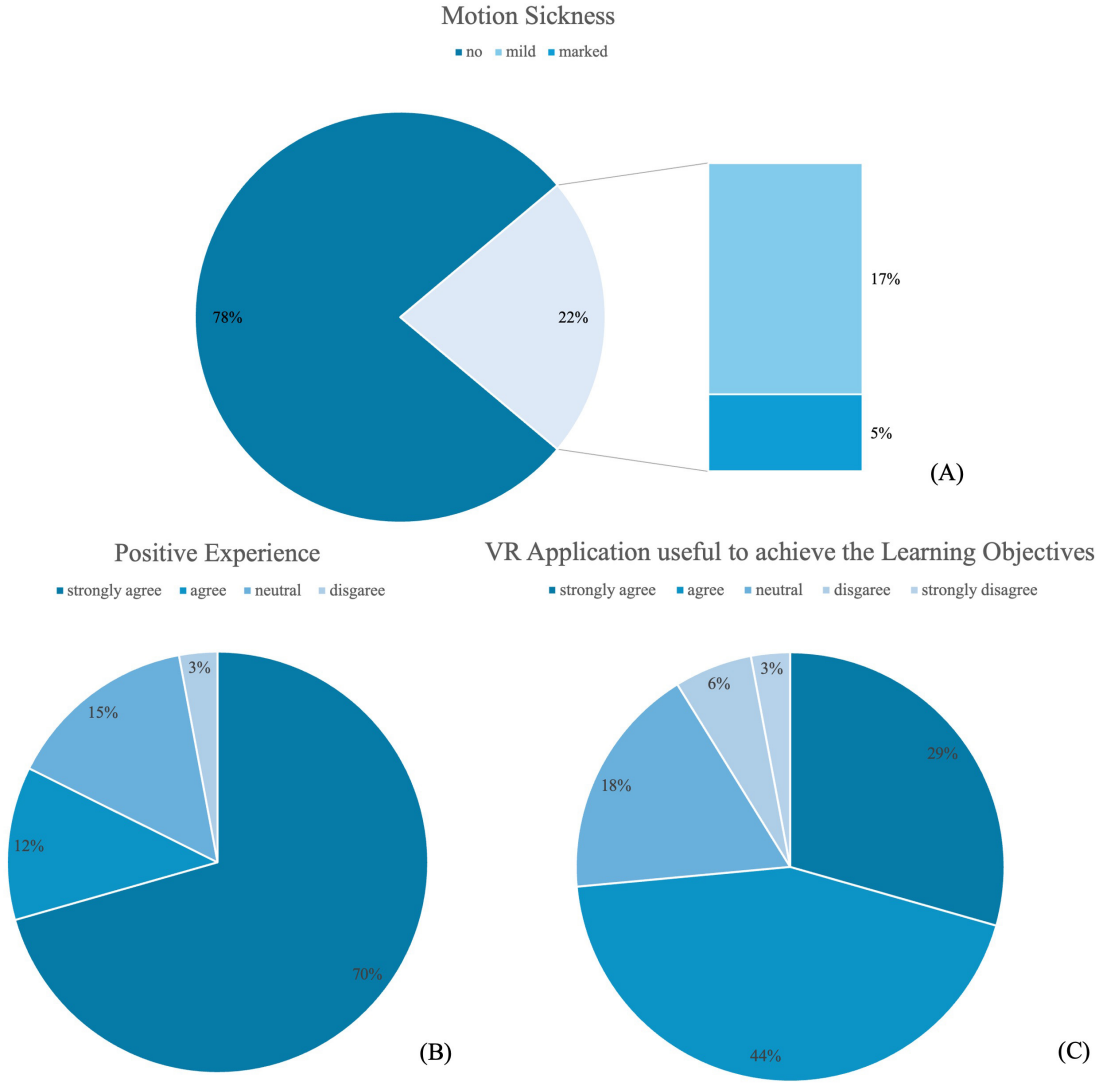
Distribution of responses regarding the VR application. **(A)** Motion sickness symptoms during VR application [responses 1–5 collapsed into “no symptoms” (1–3), “mild symptoms” (4), and “marked symptoms” (5)]. **(B)** Positive evaluation of the VR application. **(C)** Perceived usefulness of the VR application for achieving learning objectives.

With regard to subjective evaluation, participants reported low levels of motion sickness (median = 2 [IQR 1–3]) and rated the VR application positively (median = 5 [IQR 4–5]) and as a useful tool for achieving learning objectives (median = 4 [IQR 3–5]) on the 5-point Likert scale (from 1 strongly disagree to 5 strongly agree), as demonstrated in Table 5 and Figures 10A–C.

**Table 5:** Descriptive statistics for subjective evaluation items (5-point Likert scale).

**Item**	***N***	**Median [IQR]**	**Mean + SD**	**Minimum**	**Maximum**
I experienced motion sickness during the application.	35	2 [1–3]	2.26 + 1.31	1	5
I perceived the virtual reality application as positive.	34	5 [4–5]	4.5 +0.86	2	5
The VR technology was useful for achieving the learning objectives.	34	4 [3–5]	3.91 +0.996	1	5

Values are reported as median [interquartile range] and mean ± standard deviation.

Specifically, 27 participants reported no motion sickness (14 strongly disagree, eight disagree, five neutral), while 10 indicated some degree of motion sickness, of whom eight reported mild (agree) and two marked symptoms (strongly agree). Regarding the evaluation of the application, 28 students expressed a positive experience (24 strongly agree, four agree), five were neutral, and only one gave a negative rating (disagree; none selected strongly disagree). Similarly, when asked about the usefulness of the VR technology for achieving the learning objectives, the majority responded positively, with 25 students agreeing (15 agree, 10 strongly agree), while six remained neutral and three expressed disagreement.

Free-text responses highlighted aspects such as the innovative character of the teaching method, the immersive 3D 360° visualization of the topic (very frequently mentioned), and the opportunity to use VR within the university setting. Concrete suggestions for improvement mainly referred to the integration of additional pathological cases, the addition of audio content for reinforcement, the possibility of interaction with the patient and a more realistic programming of the delivery room environment.

## 4 Discussion

The present study evaluated the effectiveness and acceptance of a novel VR-based module (V.T.O.B.S.) designed to teach the mechanics of childbirth within the undergraduate medical curriculum. While VR is increasingly used in medical education and several studies in obstetrics have demonstrated promising effects on knowledge, skills, and learner confidence, evidence remains heterogeneous and long-term outcomes are rarely assessed (15, 17, 22). Against this backdrop, our findings provide important insights into the role and limitations of VR in obstetrics education and allow a nuanced interpretation in light of existing literature.

In this study, no statistically significant difference in long-term (>4 weeks) knowledge gain was found between the VR and the control groups. This finding was unexpected, as immersive visualization of birth mechanics was hypothesized to provide a measurable advantage in the summative assessment. Students in their ninth semester also tended to score higher, which may be attributed not only to greater clinical experience but also to the shorter interval between block internship and OSCE examination. In contrast, eighth-semester students completed the internship one semester earlier, resulting in a substantially longer gap between teaching and assessment. Importantly, VR did not perform worse than the traditional seminar format. Students in the VR group achieved knowledge outcomes comparable to those in the seminar group, indicating that immersive technology can provide an equivalent level of knowledge transmission while at the same time offering added value in terms of acceptance, usability, and the visualization of hidden 3D processes.

Subgroup analyses revealed no significant differences by gender, prior VR or OB/GYN experience, or learning style, suggesting that immersive learning was perceived similarly across profiles. Larger, balanced samples are needed to clarify potential moderating effects.

Acceptance and usability of the VR application were consistently rated very high, with motion sickness being rare and mild, confirming its tolerability. Participants emphasized the immersive visualization and novelty of the module and proposed additions such as pathological cases or audio reinforcement. These findings confirm feasibility and align with prior reports of high acceptance of VR in medical education (10, 23, 24).

However, this positive perception was not accompanied by significantly higher knowledge scores in the long-term assessment. Possible explanations may include the single, self-directed exposure, the complexity of the content, and the limitations of the theory-based OSCE station in capturing deeper spatial understanding. This underscores the need for carefully chosen outcome measures in future research. Beyond the statistical interpretation, it is important to distinguish between the absence of significant quantitative effects and the potential educational relevance of the intervention. Although no measurable difference in OSCE scores was observed, qualitative and observational findings suggest that the VR module may have fostered engagement, spatial understanding, and intrinsic motivation. Participants' free-text comments frequently emphasized the immersive and clarifying nature of the visualization, indicating a perceived enhancement of conceptual learning. Such subjective and attitudinal benefits, even without corresponding statistical improvement, may still reflect meaningful educational value and justify further exploration of VR-based approaches under optimized learning conditions.

When compared with existing literature, our findings both align with and diverge from previous work, depending on the specific learning objectives and study designs. Several randomized controlled trials in obstetrics and gynecology have shown that VR interventions can significantly enhance knowledge or performance, particularly in narrowly focused scenarios. For example, Kim et al. (12) demonstrated higher confidence and knowledge scores in VR-based training for premature rupture of membranes management and cesarean section, while Falcone et al. (13) reported superior adherence to algorithms and faster delivery times in a shoulder dystocia emergency simulation. Similarly, McEvoy et al. (14) found that VR-trained residents performed uterine balloon tamponade more accurately and efficiently than controls. Most recently, Hüseyinoglu and Yazici (18) reported that a VR-based labor mechanism application improved not only immediate knowledge but also retention over time, further underscoring the potential of immersive training in obstetrics (18). These studies highlight VR's potential to improve targeted procedural or emergency-related skills, particularly under conditions of repeated exposure and standardized assessment.

The present results thus complement a growing body of contemporary evidence (2023–2025) confirming the pedagogical versatility of immersive training across obstetric scenarios. In addition to procedural benefits, recent systematic reviews have emphasized the importance of assessing higher-order cognitive and psychomotor outcomes in VR learning environments (9, 25, 26). By situating our findings within this evolving literature, the study contributes to defining realistic expectations for single-session VR interventions in undergraduate curricula.

In contrast, other investigations more comparable to our approach did not identify significant performance benefits despite consistently positive student evaluations. Ryan et al. (24), for example, compared a short VR learning environment on fetal development with a PowerPoint-based session and observed no significant differences in knowledge gain, although students reported higher satisfaction with VR. Ellington et al. (27) similarly found no added benefit of immersive VR anatomy models over traditional study curricula among OB-GYN residents, despite strong subjective endorsement of the VR format. In the context of obstetric teaching, such as in the present study, these investigations were characterized by brief or single exposures, broader learning targets, and outcome measures emphasizing factual recall rather than conceptual understanding, which may explain the lack of significant effects.

However, immersive use may also reduce instructor interaction, increase cognitive load, or distract from learning objectives, emphasizing the need for blended formats that combine VR's visual strengths with guided teaching. Traditional seminars may confer short-term advantages in summative testing due to immediate feedback and focused group dynamics. Given the single, self-directed exposure in our study, such factors likely contributed to comparable outcomes despite strong acceptance of the VR module.

Several factors may account for our findings. A central aspect concerns the learning target. The V.T.O.B.S. module is unique in that it provides a broad and comprehensive visualization of both physiological and pathological birth mechanics. Such holistic and conceptual content is inherently more difficult to capture in a short OSCE station with 12 questions over 5 min, which emphasizes factual recall and rapid interpretation of 2D images rather than deeper spatial understanding or mental model building. Consequently, the present assessment format may not have been sensitive enough to detect more nuanced gains resulting from immersive learning. This reflects an important limitation: while the exam was appropriate to capture knowledge on the tested dimension and showed no inferiority of VR, it was not designed to measure the very type of learning (mental model construction and 3D reasoning) that immersive technology is expected to promote. Moreover, given the curriculum-constrained sample size, the study was not powered to rule out small effects; effects below the minimum detectable threshold may have gone undetected.

In contrast, most prior VR studies in obstetrics have focused on discrete procedures or emergencies, where performance could be directly assessed using algorithm adherence or technical skills checklists. Other designs have employed knowledge quizzes, confidence ratings, or procedural checklists, which may be more sensitive to VR-related benefits. Importantly, long-term retention of knowledge and skills has rarely been investigated in VR studies to date (25, 26, 28). A recent randomized controlled trial in obstetrics demonstrated that a VR-based labor mechanism application improved not only immediate knowledge but also retention over several weeks of follow-up (18). In contrast, Gray et al. (29) found that midwifery students benefitted from 3D visualization of the third stage of labor in a post-test conducted directly after the intervention, but these advantages were no longer detectable at follow-up (29). Likewise, Essoe et al. (30) showed that VR-related knowledge gains are strongly context-dependent, with retention decreasing over time unless mental context reinstatement is provided (30). These findings mirror our own results: while V.T.O.B.S. was expected to facilitate deeper understanding through immersive visualization, no significant advantage was observed in the OSCE more than 4 weeks after the intervention.

Beyond obstetrics, evidence from other domains of health education further supports this interpretation. Liu et al. (25) demonstrated that VR can improve skill retention in nursing education, indicating that durable effects are possible under certain conditions (25). Phillips et al. (26) reinforced that many VR studies lack delayed assessments, leaving long-term efficacy uncertain (26). Taken together, these studies indicate that while VR may offer clear benefits in the immediate post-intervention phase, sustained improvements depend on reinforcement, contextual alignment, and repeated practice

Another important factor is the intensity and frequency of exposure. While studies reporting significant VR effects often implemented structured, repeated, or supervised sessions (13, 14), students in our study engaged with the module only once in a self-directed format. The available time frame was up to 2 h, but most students spent roughly 45 min, with considerable variation. In line with the concept of deliberate practice, repeated exposure and guided learning are likely required to translate VR's didactic potential into measurable performance improvements.

Finally, the study design itself may have contributed. The inclusion of both eighth- and ninth-semester students meant that intervals between intervention and assessment varied. Although a shorter interval in the VR group might have been expected to yield performance advantages, such an effect did not materialize, suggesting that additional factors, such as intensive exam preparation by all students and the single, self-directed nature of the intervention, likely outweighed any benefit of reduced time lag combined with VR training.

Beyond the absence of a VR-related effect, one consistent and robust factor influencing performance was visual impairment. Students with visual impairment consistently scored lower in the OSCE compared to those without impairment. This indicates that the observed disadvantage is not specific to VR but reflects a broader challenge in mastering visually and spatially demanding tasks. OSCE items on birth mechanics, in particular, require strong 3D visualization skills. Prior research supports this interpretation, demonstrating that spatial ability is a key determinant of performance in anatomy and surgical education (19, 31, 32). Thus, visual impairment may compromise students' ability to form accurate mental models of complex spatial processes, underscoring the need for tailored didactic support such as tactile models, adaptive technologies, or repeated scaffolded VR exposure. Complementary non-immersive formats (e.g., desktop-based simulations or interactive videos) may provide alternatives in these cases and strengthen inclusivity in medical education more broadly.

Despite these limitations, V.T.O.B.S. should be considered innovative compared with most prior VR applications. Whereas, many existing interventions concentrate on narrowly defined skills or emergency algorithms, V.T.O.B.S. provides a comprehensive and immersive 3D 360° visualization of both physiological and pathological birth mechanics. This breadth makes it one of the first attempts to translate an entire obstetric process, rather than isolated procedures, into an interactive virtual learning environment. By combining normal and abnormal mechanisms of labor, the application addresses a curricular gap that is often difficult to cover in conventional teaching due to limited clinical exposure and ethical considerations. Beyond its content, V.T.O.B.S. also represents an important step in the integration of immersive technology into undergraduate obstetrics curricula and highlights its potential not only for undergraduate students but also for postgraduate training and interprofessional education.

From a didactic perspective, VR offers clear advantages in visualizing hidden, dynamic 3D processes such as fetal rotation and descent, which are difficult to convey through static models or instructor demonstrations alone (10). By supporting the development of accurate mental models and reducing cognitive load (4, 6, 8–10), VR can enhance understanding of complex spatial content. These processes align with theoretical models such as the Cognitive-Affective Model of Immersive Learning (CAMIL), which links immersive media features to attention, emotion, and cognitive engagement (33). Beyond its use in obstetrics, the technology holds potential for interprofessional learning, particularly as this module was developed jointly with the midwifery faculty, highlighting the relevance of VR for collaborative training in maternal care.

Taken together, this study demonstrates that while measurable short-term performance gains could not be confirmed, the consistently positive evaluation and the unique didactic potential of V.T.O.B.S. provide a strong rationale for further investigation and optimization of VR-based learning in obstetrics.

This study has several limitations. It was conducted at a single center and included unequal group sizes, which may limit generalizability and statistical power. The VR module was used only once and in a self-directed format without standardized time requirements, leading to variability in individual engagement. The interval between intervention and OSCE assessment was long and heterogeneous (8–14 weeks), and intensive exam preparation by all students likely reduced group differences. Moreover, using both eighth- and ninth-semester students in the control group may have introduced subtle inter-cohort differences related to timing, examiner variation, or student motivation. While these effects are unlikely to have systematically favored one group, they represent potential confounders that should be acknowledged when interpreting the findings. Another limitation concerns the use of unvalidated self-developed evaluation items, which may have influenced measurement reliability and comparability across studies.

Future research should aim to build on these findings by implementing repeated and longitudinal VR sessions to strengthen learning effects and assess knowledge retention over time. More granular, item-level analyses, particularly of image-based tasks that rely heavily on spatial visualization, may provide additional insights into which competencies benefit most from immersive learning. Larger, multicenter trials will be needed to confirm the generalizability of these results and to explore the role of VR across diverse curricular contexts. Moreover, future work should not be limited to undergraduate students, but also include midwifery students, physicians in training, practicing clinicians, and certified midwives will be essential to evaluate the potential of VR across different stages of professional development. Finally, the integration of VR into interprofessional training formats involving both medical and midwifery learners represents a promising avenue to foster collaboration and shared understanding in obstetric care.

In practical terms, the strong acceptance and feasibility of V.T.O.B.S. support its integration into existing curricula as a complementary, self-directed or blended-learning module. Multi-institutional and longitudinal studies should next evaluate repeated use, effects on long-term knowledge retention, and transfer to clinical or interprofessional performance to guide sustainable VR implementation in medical education.

In conclusion, the VR application V.T.O.B.S. represents a feasible and well-accepted innovation for teaching birth mechanics in undergraduate medical education. While no significant improvement in long-term knowledge gain was demonstrated in our study, learners evaluated the module positively and reported high usability with minimal side effects. These findings indicate that VR may offer added value as a complementary teaching tool, particularly through its ability to visualize hidden, dynamic 3D processes and to support interprofessional learning. At the same time, the absence of measurable performance benefits in our long-term assessment highlights the need for cautious interpretation and reflects the heterogeneous evidence reported in the literature. Further research is therefore required to determine under which conditions VR can translate its high acceptance and didactic potential into durable learning outcomes.

## Data Availability

The datasets generated and analyzed during the current study are available from the corresponding author upon reasonable request.
